# Sustained release of decorin to the surface of the eye enables scarless corneal regeneration

**DOI:** 10.1038/s41536-018-0061-4

**Published:** 2018-12-21

**Authors:** Lisa J. Hill, Richard J. A. Moakes, Chairut Vareechon, Gibran Butt, Aaron Ng, Kristian Brock, Gurpreet Chouhan, Rachel C. Vincent, Serena Abbondante, Richard L. Williams, Nicholas M. Barnes, Eric Pearlman, Graham R. Wallace, Saaeha Rauz, Ann Logan, Liam M. Grover

**Affiliations:** 1 Institute of Clinical Sciences, University of Birmingham, Birmingham, B15 2TH UK; 2 School of Chemical Engineering, University of Birmingham, Birmingham, B15 2TT UK; 3 Department of Ophthalmology, University of California, Irvine, CA 92612 USA; 4 Institute of Inflammation and Ageing, University of Birmingham, Birmingham, B15 2TT UK; 5 Birmingham and Midland Eye Centre, SWBH NHS Trust, Birmingham, B18 7QH UK; 6 Diagnostics, Drugs, Devices and Biomarkers clinical trials team (D3B), Institute for Translational Medicine, University of Birmingham, Birmingham, B15 2TT UK

## Abstract

Disorganization of the transparent collagenous matrix in the cornea, as a consequence of a variety of infections and inflammatory conditions, leads to corneal opacity and sight-loss. Such corneal opacities are a leading cause of blindness, according to the WHO. Public health programs target prevention of corneal scarring, but the only curative treatment of established scarring is through transplantation. Although attempts to minimize corneal scarring through aggressive control of infection and inflammation are made, there has been little progress in the development of anti-scarring therapies. This is owing to eye drop formulations using low viscosity or weak gelling materials having short retention times on the ocular surface. In this study, we report an innovative eye drop formulation that has the ability to provide sustained delivery of decorin, an anti-scarring agent. The novelty of this eye drop lies in the method of structuring during manufacture, which creates a material that can transition between solid and liquid states, allowing retention in a dynamic environment being slowly removed through blinking. In a murine model of *Pseudomonas* *keratitis*, applying the eye drop resulted in reductions of corneal opacity within 16 days. More remarkably, the addition of hrDecorin resulted in restoration of corneal epithelial integrity with minimal stromal opacity endorsed by reduced α-smooth muscle actin (αSMA), fibronectin, and laminin levels. We believe that this drug delivery system is an ideal non-invasive anti-fibrotic treatment for patients with microbial keratitis, potentially without recourse to surgery, saving the sight of many in the developing world, where corneal transplantation may not be available.

## Ophthalmology: novel eye drop brings sustained drug delivery to ocular surface

An eye drop formulation that applies anti-scarring drugs to the surface of the eye helps reverse infection-induced corneal damage in mice. Hill et al. from the University of Birmingham, UK, formulated a fluid gel loaded with a wound-healing protein called decorin that conforms to the ocular surface and is cleared gradually through blinking. With colleagues in California, they applied the therapeutic eye drop to mice with bacterial eye infections that trigger sight-threatening corneal scarring. Within a matter of days, the team saw improvements in corneal transparency, with reductions in scar tissue and reconstitution of healthy cells. Such a drug delivery system, if successful in humans, could help save many people’s sight and reduce the need for corneal transplantation.

## Introduction

Corneal opacity is a leading cause of sight impairment worldwide with an estimated 27.9 million people globally being bilaterally or unilaterally affected.^1^ Such opacity is typically derived from alteration of the complex, optically clear, corneal tissue structure, vital for refraction of light onto the retina, and subsequent neuro-visual processing. Commonly, corneal scarring results from ocular infections from a range of pathogens, including bacteria, parasites, fungi, viruses, and protozoa. In the developed world, devastating corneal infections are most commonly associated with prolonged contact lens wear and/or poor lens hygiene,^2–4^ with *Pseudomonas aeruginosa* being a prominent causative organism. In cases of gram-negative infections, e.g., *Pseudomonas*, the structural integrity of the cornea becomes compromised through multiple virulence factors, whereby the microbes invade epithelial cells, resulting in activation of numerous inflammatory pathways. Subsequent production of cytokines from epithelial, stromal and intraepithelial inflammatory cells, neovascularization, cellular alterations and degradative stromal processes^5^ lead to dysregulated tissue remodeling and disruption of the intricately arranged collagen fibrils,^6^ leading to the loss of optical transparency, impairment of light refraction and loss of sight.

Following injury that breaches the epithelium and Bowman’s layer involving the corneal stroma, an orchestrated wound healing response involving corneal epithelium, stroma and nerves, lacrimal glands and tear film occurs to restore corneal structure and function and maintain the ocular integrity.^7^ As part of the corneal wound healing response, the epithelium starts to regenerate in response to stem cell proliferation from the limbal niche almost immediately after the epithelium is injured^8^ and keratocytes (transparent cells that function to maintain collagen and extracellular matrix (ECM) turnover) proximal to the wounding site undergo apoptosis (induced by cytokines released from damaged epithelial cells). Proliferation and migration of residual keratocytes peripheral to the injury-site can be detected 12 to 24 h after injury.^9^ The keratocytic response includes production of proteoglycans and synthesis of collagen fibers. These fibers are larger than those in an uninjured cornea and, owing to the water-retention capacity of the proteoglycans, do not assume an ordered regular architecture leading to corneal opacity. The movement of bone marrow-derived precursor cells and circulatory mediators from the limbal region activate, transform, and differentiate a subset of keratocytes to cell types with fibroblast and myofibroblast characteristics via Transforming Growth Factor (TGF)β and platelet-derived growth factor (PDGF) activation.^9–11^ TGFβ released from epithelial cells access stromal cells through the damaged Bowman’s layer to initiate myofibroblast differentiation.^12,13^ Myofibroblasts release cytokines that further attract inflammatory cells and ECM deposition (e.g., collagen, fibronectin) to facilitate the fibroblast migration as part of the stromal remodeling phase.^14^ Reparative processes lead to collagen fiber orientation closer to that in the uninjured cornea, contraction of proteoglycan, apoptosis of the stromal myofibroblasts, and re-population of keratocytes enable structural and functional recovery, but with residual stromal opacity. If this is present in the visual axis, sight is impaired. If the corneal epithelial barrier is not restored, stromal metabolism becomes dysregulated leading to keratolysis, degradation of corneal tissue, further disorganization of corneal fibril arrangements, and eventual corneal perforation. This is partly due to the sustained release of TGFβ leading to persistent myofibroblasts preventing stromal re-population of keratocytes.^15,16^ Unlike other tissues (e.g., skin), where a persistent ulcer or scar might be tolerated, in the cornea this can have devastating functional effects of permanent corneal scarring with visual disability or loss of eye.

At present, the standard of clinical care for patients infected with bacterial keratitis focuses initially on sterilizing the infected eye, by eye drop administration of intensive broad-spectrum antibiotics, followed by the addition of topical corticosteroids to reduce inflammation.^17,18^ This is followed by strategies to limit scar formation ranging from intensive lubrication (to reduce biomechanical trauma of the eyelids abrading the wound bed during blinking), to the use of systemic pharmacological agents (sub-antimicrobial dose of tetracyclines for matrix metalloproteinase inhibition^19^) or supplements (vitamin C used as antioxidants and free-radical scavengers^20^) in an attempt to promote tissue remodeling. Unfortunately, although effective at sterilizing the eye, the patient is often left with a high degree of corneal hazing and astigmatism which, if it compromises the visual axis, causes loss of visual acuity. Surgical interventions to treat unresponsive and large corneal defects include either application of amniotic membrane as a biologically active bandage releasing anti-inflammatory and anti-fibrotic factors to enhance re-epithelialization and wound healing during acute injury,^21–23^ or in established cases of visually significant central corneal scars, excision of the scarred tissue, and replacement with donor cornea. Reproducibility and repeatability of the clinical outcomes of amnion grafting and corneal transplantation are low and fraught with risks of failure and rejection.^24–27^ Enhancement of re-epithelialization, prevention of keratolysis, and minimization of fibrosis are pivotal to effective wound healing, mitigating the need for surgical intervention and transplantation. An innovation that could saturate TGFβ activity^28–30^ and provide an exogenously placed barrier to enable a microenvironment that minimizes corneal damage following the injury response, could have the potential to prevent permanent sight-loss in many millions of individuals worldwide.

Decorin is a naturally occurring, pleiotropic, small leucine-rich proteoglycan that is naturally present at high levels bound to collagen in the corneal stroma and which, when released, tightly regulates TGFβ activity by binding the growth factor and sequestering it within the ECM.^31^ Decorin regulates cell proliferation, survival, and differentiation by modulating numerous growth factors,^32–35^ including TGFβ as well as directly interfering with collagen fibrillogenesis.^36–39^ Decorin is responsible for regulating collagen fibril spacing and ECM to enable corneal transparency and has previously been shown to inhibit scar formation and neovascularization in the cornea.^35^ Mutations in decorin are associated with corneal opacities and visual abnormalities associated with congenital stromal dystrophy.^40^ Hyperactivity of TGFβ in corneal fibrosis may overcome the ability of endogenous decorin to maintain homeostasis and there is good evidence that overexpressing decorin in other tissues is able to reduce levels of fibrosis in vivo.^41–43^

Human recombinant (hr)Decorin is now available in GMP form and this same molecule has been shown to minimize fibrosis in other in vivo models of brain and spinal cord injury.^44–46^ To date, there has been no reported efficacy of soluble decorin being applied to the surface of the eye for treatment in vivo. One of the possible reasons for this maybe the relatively rapid clearance of eye drops (in the range of minutes^47,48^), owing to their relatively low viscosities, from the surface of the cornea at early time points, meaning that any efficacy of decorin would be limited. In this paper, we report a new class of eye drop material that allows for prolonged retention of a therapeutic on the surface of the eye, while being gradually cleared through the blinking process. The material is formed through the shearing of a gellan-based hydrogel, a material that is currently used in dilute form to thicken eye drops (e.g., Timoptol) during the gelation process. The application of shear prevents the formation of a continuous polymeric network and results in the formation of interacting particles that can exhibit spherical and ribbon-like morphology. Following shear-processing, these particles interact and form a continuous structure when the solution is at rest. When shear is applied (such as when extruded through an eye dropper), however, the continuous network of particles is disturbed and the material liquefies. Subsequent removal of the shear force results in an immediate healing. The solid–liquid–solid transitions that this material is able to undergo means that it conforms perfectly to the ocular surface and is removed gradually by the eyelid-blinking dynamics. Importantly, gellan gum is optically transparent and so the material can continue to transmit light following application, causing minimal disruption to the patient.

We have developed a fluid gel eye drop, which can be loaded with decorin, to provide localized drug delivery and retention at the surface of the eye. The material combines structured gellan gum with the proteoglycan, decorin. Additionally, in conjunction to high optical clarity, the FDA approved polymer (FDA reference number 172.665) coupled with clinical grade hrDecorin, provides a rapid route to translation into the clinic. As such, this study investigated the effects of fluid gel, with and without hrDecorin, on corneal opacity, wound healing and fibrosis within a well-established murine model of *Pseudomonas keratitis*, as a precursor to clinical application for the management of severe bacterial infection.

## Results

### Fluid gel formulation and properties

**Fig. 1:**
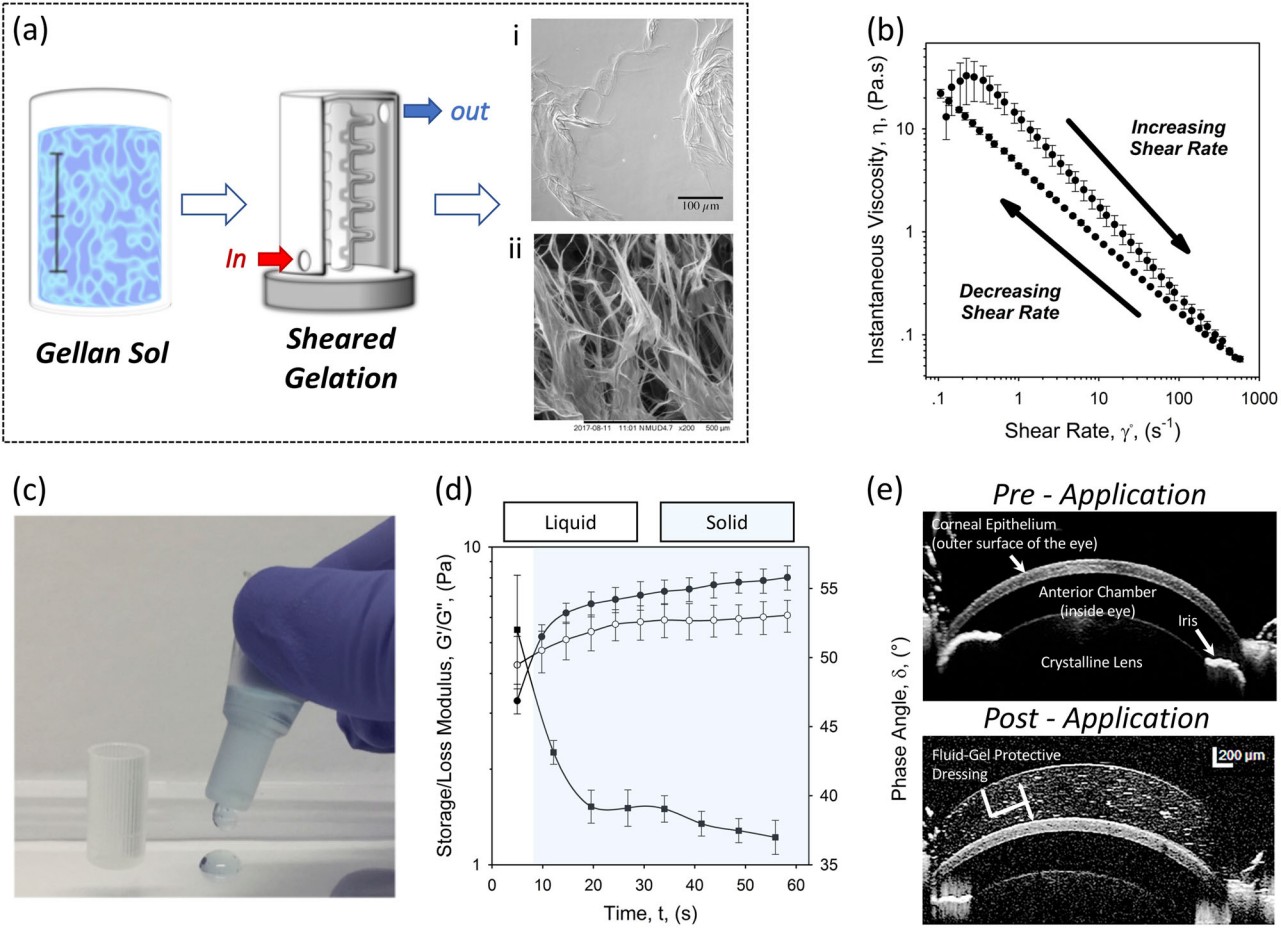
Processing and intrinsic material properties of the gellan-based fluid gel eye drop. **a** Schematic showing the production of the fluid gel: where the initial sol is continuously processed under shear whilst being cooled to form “ribbon-like” gelled entities shown using (i) transmission microscopy and (ii) scanning electron microscopy. **b** Time-dependent viscosity profiles obtained for the gellan eye drop, highlighting a degree of thixotropy. **c** The fluid gel being dispensed from the eye dropper packaging (gel has been stained blue so as to be visible in the photograph). **d** Small deformation rheology data obtained at a single frequency (1 Hz, 0.5% strain) as a function of time. Data show the evolution of an elastic network post-shearing resulting in a transition from liquid to solid-like behavior. **e** Anterior segment OCT images showing the ocular surface before fluid gel application (top image) and post application (bottom image). Images demonstrate a uniform layer that covers the entirety of the ocular surface

Processing of the fluid gel involves passing a polymer solution, gellan, through a jacketed pin-stirrer, where it experiences high levels of shear while being forced (thermally) through its sol–gel transition (Fig. 1a). This restricts the long-range ordering normally observed in the formation of quiescent gels, restricting growth of the gel nuclei to discrete particles.^49^ The microstructures within the eye drop prepared in this way have been shown using two techniques: (1) optical microscopy, whereby the refractive index of the continuous phase was manipulated using polyethylene glycol, and (2) lyophilizing in order to image using scanning electron microscopy (SEM) (Fig. 1a(i) and 1a(ii), respectively). Both microscopic techniques highlight the stranded microstructure of the resulting gelled entities, where their large length to width ratio and subsequent large hydrodynamic radii, give rise to the resulting material properties (viscosity and elastic structuring).^50^

The unique properties of fluid gels are such that they exhibit pseudo-solid properties at rest, but can be made to flow under force. Here, increasing the shear force exerted on the system results in non-Newtonian, shear thinning behavior, typical of highly flocculated or concentrated polymer dispersions/solutions^51^ (Fig. 1b). As such, at low shear, large viscosities exceeding several orders of magnitude higher than typical water-based eye drops are observed, thinning during application and subsequent blinking as a result of dis-entanglement and alignment of particles in flow.^52,53^ This makes the microgel suspension ideal for application through dropper bottles, rapidly shear thinning through the nozzle on application to the eye (Fig. 1c). Post application, restoration of the three-dimensional structural matrix is key to gaining high retention times upon the ocular surface. Time-dependent removal of shear, at comparative timescales to the initial ramp, was used to gather information regarding such structuring, probing eye drop hysteresis. The eye drop system showed a degree of thixotropy (Fig. 1b), whereby the majority of the original viscosity was recovered. The presence of weak interactions between gel-ribbons were examined using linear rheology, with the evolution of an elastic structure at strains within the linear viscoelastic region (Fig. 1d). It was observed that initially, post-shear, the fluid gel exhibited typical liquid-like behavior with the loss modulus (G”) dominating the storage modulus (G’). Following this, an increase in G’ as a function of the formation of interactions between gelled ribbons led to a crossover being reached, at which point the system began to behave as a solid–gel.^54^ Further structuring over time thus leads to pseudo-solid behaviors, where a continuous network is formed between the gelled entities. The ability to shear-thin on application, while being able to quickly restructure post shearing, enables the eye drop to be applied to the ocular surface, acting as a barrier. Using a single 5 µl application of fluid gel eye drop, it was demonstrated that a uniform distribution of gel covers the entire ocular surface including cornea, adjacent conjunctiva and fornices (space between the eyelid and eyeball) in a rodent eye (Fig. 1e).

### In vitro eye drop activity

**Fig. 2:**
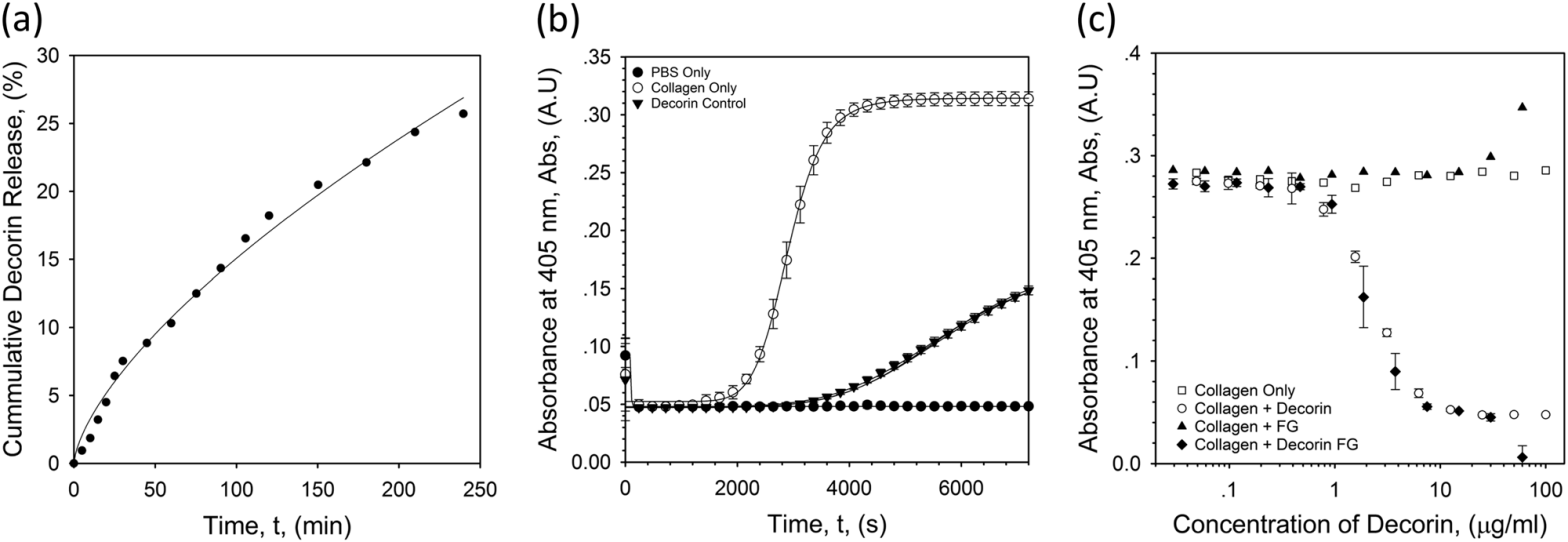
In vitro assays demonstrating the formulated eye drop’s bioactivity. **a** Cumulative release curve for hrDecorin loaded eye drops over 4 h (240 min). Line of best fit follows a power function, *y*=0.7*x*^0.7^ (*R*^2^ = 0.99). **b** Collagen fibrillogenesis turbidity data for PBS control, collagen only and collagen + hrDecorin. **c** Collagen fibrillogenesis turbidity data with a dose response curve for collagen, collagen + hrDecorin, collagen + fluid gel (FG) only, collagen + hrDecorin loaded fluid gel (DecFG)

The gellan-based eye drop system was formulated for drug delivery with the candidate anti-fibrotic agent, hrDecorin, used for our studies. The rate of release of hrDecorin from the eye drop system was almost linear over time (Fig. 2a). Turbidity was used as a measurement of fibrillogenesis (formation of large, disorientated collagen fibers), shown as a function of the hrDecorin (Fig. 2b, c). It was evident that hrDecorin played a key role in the kinetics of fibril formation, slowing the onset of fibrillogenesis, and also reaching an equilibrium much faster (Fig. 2b). Above a critical concentration, 0.5 μg/ml, an active effect of hrDecorin in inhibiting fibrillogenesis was observed, highlighting a concentration dependency until a minimum turbidity is achieved (> 10 μg/ml), above which no further reduction occurs (Fig. 2c). Furthermore, the assay demonstrated that the fluid gel carrier had no effect on fibril formation, correlating closely with the collagen only controls.

### In vivo efficacy of the loaded/unloaded eye drop on corneal opacity

Using a well-established model of bacterial keratitis,^55^ anaesthetized mice (*n* = 6 per group) were challenged with *P. aeruginosa* (10^5^ colony-forming unit; CFU) upon the surface of the damaged cornea. A therapeutic protocol to treat the infection based upon the standard treatment for bacterial keratitis patients was developed. After 12 h of *P. aeruginosa* incubation to establish the corneal infection, eyes were treated with a 2-hourly regime of gentamicin (1.5%) over a 12-hour period to sterilize the infection (confirmed by swab cultures). Following the sterilization phase, 2 days after the initial inoculation, single 5 µl gellan eye drops were administered every 4 h between 8 am and 8 pm for a further 13 days covering the treatment groups of (1) Gentamicin and Prednisolone (G.P); (2) Gentamicin, Prednisolone, and fluid gel (G.P.FG) and; (3) Gentamicin, Prednisolone, and hrDecorin fluid gel (G.P.DecFG) (Table 1).

**Table 1:** Table of treatment groups

			Therapeutic	
Group no.	Gentamicin	Prednisolone	Fluid gel (FG)	hrDecorin + Fluid gel (DecFG)
1	+	+	**−**	**−**
2	+	+	+	**−**
3	+	+	**−**	+

Table highlighting the combination of therapeutics administered (+) or not administered (**−**) to each group, *n* = 6.

No evident systemic toxicity was present in mice after treatments and no systemic exposure to decorin was detected from serum (Supp. Figure 1). The fluid gel and hrDecorin fluid gel were all tested in accordance with ISO10993 biocompatibility standards, tests were undertaken by an accredited Contract Research Organisation. Both of the formulations passed with direct toxicity assessments failing to identify any toxicity associated with the fluid gel or the hrDecorin fluid gel in the ISO battery of in vitro and in vivo assays.

**Fig. 3:**
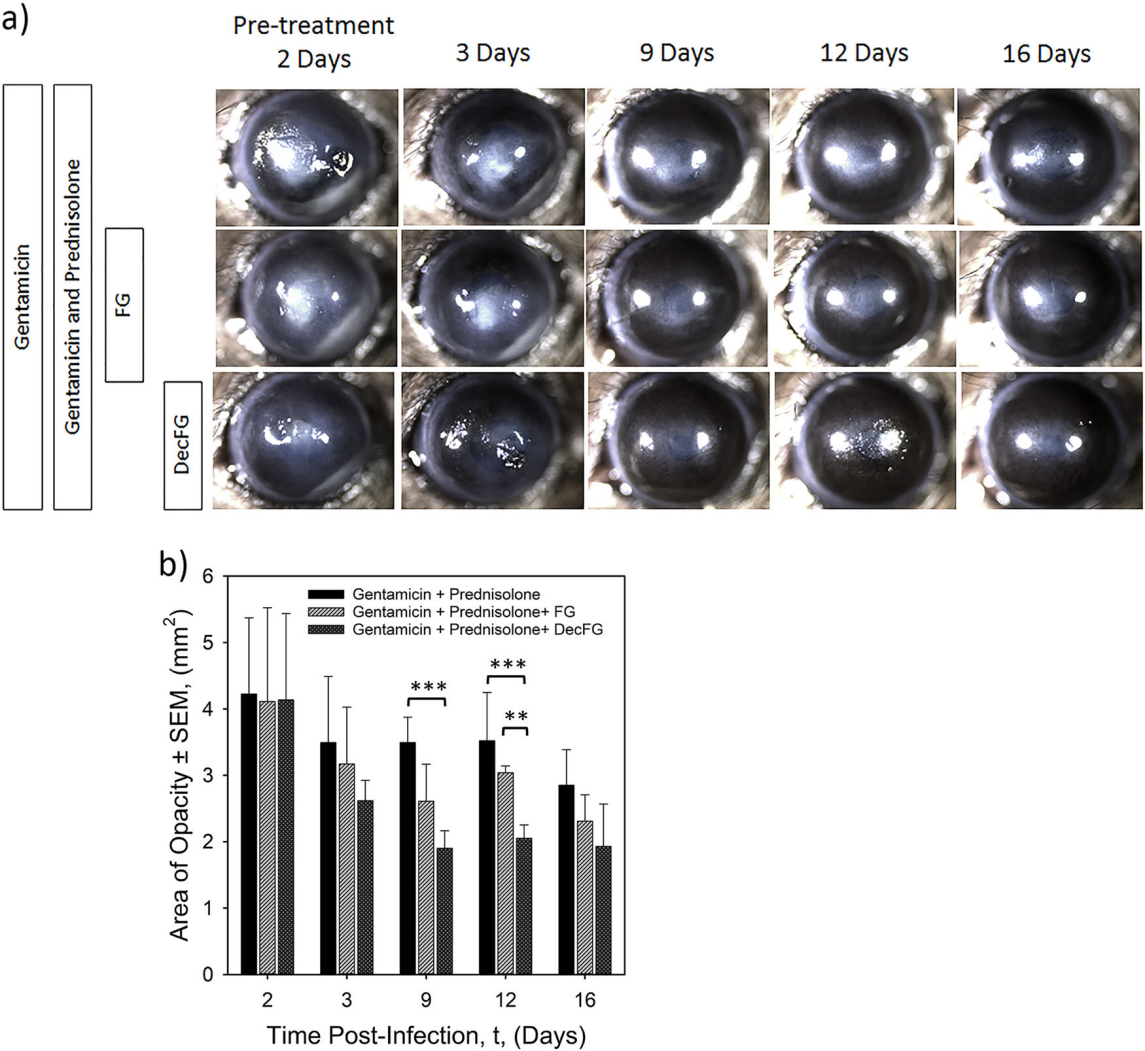
Corneal opacity area measurements. **a** Representative photographs taken at days 2, 3, 9, 12, and 16 post *Pseudomonas* infection and treatment. **b** Graph to show the mean area ± SEM (mm^2^) of opacity as measured by two independent masked ophthalmologists from photographs (represented in **a**) taken from each individual mouse per group (*n* = 6; **p < 0.01, ***p < 0.001)

Images of the cornea were taken at intervals throughout the 16-day experiment to measure changes in corneal opacity (Fig. 3a). All mice were euthanized on day 16. The area of opacity (measured independently by two clinical ophthalmologists, masked to treatment groups) showed earlier size-reduction in eyes treated with the fluid gel and with the hrDecorin fluid gel eye drops plus standard of care compared with eyes treated with standard of care (Gentamicin and Prednisolone) treatments alone. Accordingly, at day 9, eyes treated with the standard of care with hrDecorin fluid gel, showed significantly (*p* < 0.001) lower opaque areas (1.9 ± 0.3 mm^2^) compared with eyes treated with Gentamicin and Prednisolone only (3.5 ± 0.4 mm^2^). At 12 days, the mice that received hrDecorin fluid gel eye drops with standard of care maintained significantly lower (*p* < 0.01) opaque area compared with the Gentamicin and Prednisolone group, and also to the fluid gel group with standard of care (mean opacity area in Group 1 = 3.5 ± 0.7 mm^2^, Group 2 = 3.0 ± 0.1 mm^2^, in comparison to Group 3 = 2.1 ± 0.2 mm^2^; Fig. 3b).

### Effects of the fluid gel eye drops with and without hrDecorin on corneal re-epithelialization

**Fig. 4:**
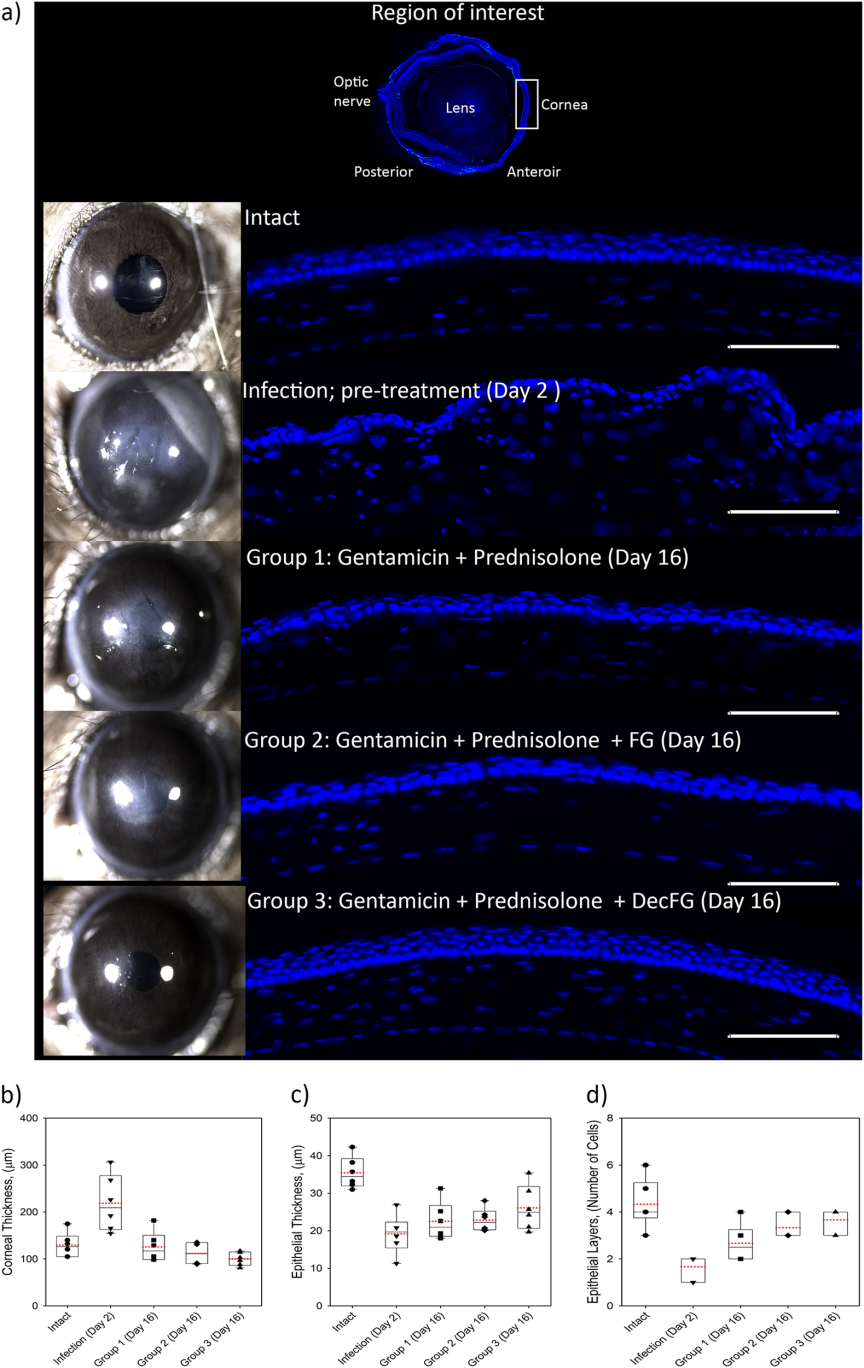
Corneal re-epithelialization. **a** Representative images of DAPI^+^ cell nuclei (blue) in the cornea used to assess the epithelium, illustrating the thickness and stratification (number of cell layers) of the epithelium in: naive intact eyes showing normal non-keratinized stratified (approximately five layers) epithelium; eyes taken at day 2 after infection, which was associated with a thickened edematous stroma with cellular infiltrate; and eyes taken at 16 days post treatment showing re-epithelization with a 2–3 layer stratification accompanied by a reduction in the stromal edemas in Group 1 (Gentamicin and Prednisolone), increased stratification in Group 2 (G.P.FG) and, fully mature epithelium in Group 3 (G.P.DecFG) (scale bar 100 µm). **b** Quantification of corneal thickness ± SEM. **c** Quantification of epithelial layer thickness ± SEM, and **d** Quantification of cellular epithelial stratification layers ± SEM in naive intact, (*n* = 6), with eyes evaluated at day 2, and at day 16 from each treatment group (*n* = 6 for each group). All quantification was performed on masked images unknown to the observer

Epithelial stratification/maturation together with stromal thickness were chosen as outcome measures to assess corneal re-epithelialization, and to observe thickening of the stroma from edema and cellular infiltrates (as markers of infection). *Pseudomonas* infection severely disrupted the corneal structure, with an averaged increased corneal thickness of 219 ± 24 µm after the infection on day 2 compared with naive corneal thickness values of 129 ± 11 µm. The infected corneas at day 2 had thinner epithelial layers compared with normal intact controls (19.2 ± 2.1 µm vs 35.5 ± 1.7 µm; Fig. 4a, b). With the addition of fluid gel alone and with hrDecorin eye drops treatments over 13 days it was evident that re-epithelialization was improved. Treatment with the hrDecorin loaded fluid gel eye drop led to an epithelial layer with an improved degree of stratification (26.1 ± 2.4 µm thick made up of 3.6 ± 0.2 cell layers) compared with the epithelium in the Gentamicin and Prednisolone group (22.5 ± 2.1 µm thick with 2.7 ± 0.2 cell layers) as well as the Gentamicin, Prednisolone, and fluid gel group (22.8 ± 1.3 µm thick with 3.4 ± 0.1 cell layers). However, the differences between the various groups did not reach statistical significance (Fig. 4b, c, d).

### Effects of the fluid gel on levels of myofibroblasts and extracellular matrix

**Fig. 5:**
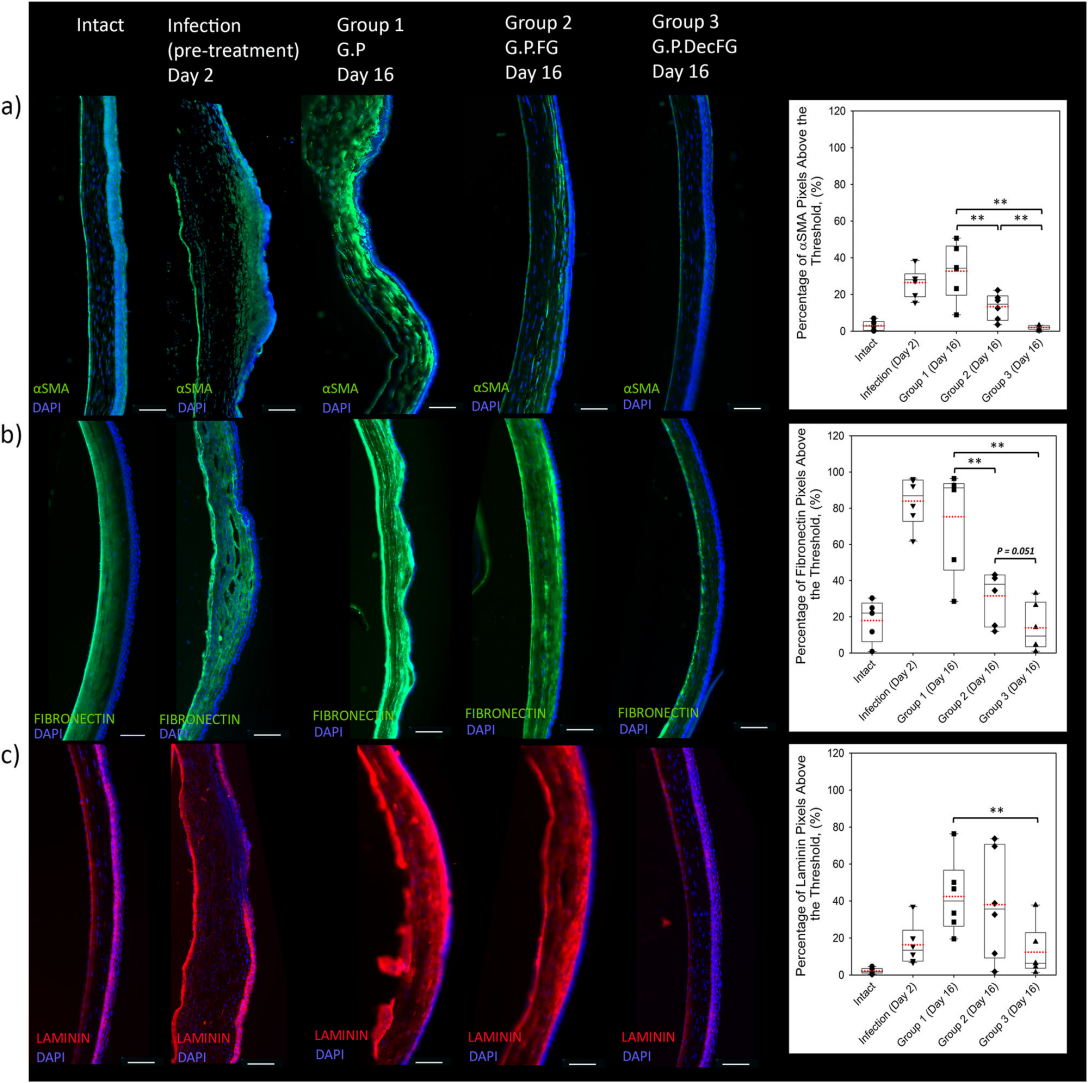
Extracellular matrix levels in the cornea. Representative images of immunohistochemical staining with accompanying plots quantifying the IR for: **a** αSMA^+^ (green to stain myofibroblasts), **b** IR fibronectin^+^ (green to stain fibronectin in the ECM), and **c** laminin^+^ (red to stain laminin in the ECM), in each case DAPI^+^ was used to stain the cell nuclei (blue). Analysis was undertaken on intact eyes, eyes taken at 2 days post infection and eyes obtained after 16 days with various eye drop treatments: (i) Gentamicin and Prednisolone (G.P), (ii) Gentamicin, Prednisolone, and fluid gel (G.P.FG), and (iii) Gentamicin, Prednisolone and hrDecorin fluid gel (G.P.DecFG). All studies were done using *n* = 6 treatment groups, with quantification performed on masked images unknown to the observer (scale bar = 100 µm)

Immunoreactivity (IR) was used to assess the degree of fibrosis, as a ratio of pixel intensity above a baseline obtained from intact corneas (referred to here on as the threshold). In the naive intact cornea, there were very low levels of α-smooth muscle actin (αSMA) IR in the corneal stroma, indicating the presence of few myofibroblasts (Fig. 5a). Two days after the infection, 1 day after sterilization, infected cornea demonstrated a 23% increase in stromal αSMA staining to levels of 26.5 ± 3.0% above the threshold (normalized from an intact cornea), indicating increased differentiation of myofibroblasts. Levels of stromal IR αSMA remained elevated at day 16, at 32.7 ± 6.1% in eyes treated with standard of care only. When eyes were also treated with the fluid gel eye drops, either with or without hrDecorin, the level of stromal αSMA IR was significantly lower at day 16, 13.4 ± 2.9% and 2.0 ± 0.4%, respectively, suggesting less myofibroblast activation within the corneal stroma. The hrDecorin fluid gel was most effective at keeping the αSMA IR levels low, resulting in similar values to the intact cornea, suggesting that the addition of hrDecorin in the fluid gel had an added beneficial effect on myofibroblast differentiation versus the fluid gel alone (Fig. 5a).

Stromal ECM levels generated by the myofibroblasts were studied using fibronectin and laminin IR (Fig. 5b, c). Increased amounts of stromal IR fibronectin were observed post infection at day 2, remaining high at day 16 after Gentamicin and Prednisolone treatment (IR Fibronectin 83.9 ± 5.5% and 75.3 ± 11.5%, at day 0 and 16, respectively). Fluid gel with or without hrDecorin, significantly lowered levels of fibronectin IR to 31.6 ± 5.8% and 13.9 ± 5.3%, respectively, demonstrating borderline significant differences (*p* = 0.051) between the two eye drop treatment groups. Levels of IR laminin (Fig. 5c) demonstrated that the infection increased levels of laminin when compared with the intact cornea, from 2.15 ± 0.6% in the intact to 16.3 ± 4.6% in the infection group at day 2. Levels of IR laminin continued to rise by day 16 after Gentamicin and Prednisolone treatment to 42.5 ± 8.2%. Similar to the Gentamicin and Prednisolone group, average levels of IR laminin remained high on day 16 after treatment with the fluid gel, with IR laminin levels at 38.0 ± 12.0%. The addition of hrDecorin to the fluid gel significantly lowered laminin levels compared with Gentamicin and Prednisolone treatment, (12.4 ± 5.5% vs 42.3 ± 8.2%), whereas the fluid gel without hrDecorin had no effect on this ECM parameter.

## Discussion

Improving ocular retention is key to increasing both bio-efficiency and therapeutic response to topical therapies, as turnover of the pre-corneal tear film (*ca*. 20% per minute^56^) results in rapid elimination of aqueous drugs, reducing titers delivered to the target tissue site. As such, many ocular conditions are currently treated via intensive topical therapies delivered through the day and night, or invasive methods disliked by many patients, including periocular or intravitreal injections to target intraocular pathology. In more severe cases where drugs are ineffective, surgery may be required to treat or remove the resultant corneal scar, increasing the risk of morbidity and increasing the duration of patient discomfort following treatment. The structured or “fluid gel” formed from gellan provides a pivotal advance since it enables the sustained delivery of molecules such as hrDecorin capable of preventing scarring and obviating the need for invasive surgical repair strategies. The major advantage of the gellan fluid gel is its capacity to transition between solid and liquid states as it passes through the applicator and solidifies on the surface of the cornea. This unique set of properties originates from the microstructure of the material, which consisted of ribbons and particles that weakly interact with one another at zero shear. These interactions are broken by the application of shear and reform following its removal. In this way, the material may then be gradually cleared from the ocular surface through the natural blinking mechanism. The development of a weak-elastic structure when applied to the surface of the cornea, results in the formation of a transparent and resorbable bandage, with the benefits of eye drops (in application) and hydrogel lens (sustained release), without the drawbacks of either. Indeed, the fluid gel alone seems to provide a m"icroenvironment conducive to wound healing, with a reduction in corneal opacity and markers of scar formation even without decorin addition. Importantly, the fluid gel does not interfere with hrDecorin’s biological activity, as shown by the collagen fibrillogenesis data. As such, the system provides an excellent candidate technology for the clinical setting, with improved drug administration compliance across numerous patient cohorts.

The mouse model of *P. aeruginosa* keratitis provides a robust, clinically relevant means of evaluating the anti-scarring capacity of the hrDecorin loaded fluid gel against the current standard of care for *Pseudomonas* infection (Gentamicin and Prednisolone).^57^ Topical administration of the fluid gel eye drops either with or without the hrDecorin resulted in reduced levels of corneal opacity after 7 and 10 days of eye drop treatments, with the addition of hrDecorin displaying an evident further advantage from earlier time points. The effects of the fluid gel only treatment were not expected as the initial in vitro studies demonstrated that this carrier appeared inert. The therapeutic efficacy of fluid gel alone may be due to the formation of a permissive microenvironment in the damaged cornea, where the occlusive effect of the gel ribbons (that entwine to form a barrier around the wound) provided a therapeutic bandage that prevented biomechanical trauma caused by blinking over the ulcerated eye. It may also have sequestered Prednisolone and Gentamicin within its structure, enhancing retention of the therapeutic substances to the ocular surface, thereby improving bioavailability similar to the prosthetic replacement of the ecosystem (PROSE^(TM)^ device) but with the added advantage of being resorbable. Such reductions in corneal opacity would benefit patients in terms of preservation of sight.^58^

An important aspect to the healing phase encompasses restoration of a stratified non-keratinized epithelium. Together with the tear film, an apical mucosa (composed of lipid, mucins and aqueous layers) provides nutrition and lubrication to the ocular surface and is fundamental to first-line of defense to the eye. hrDecorin treated eyes exhibited the most improved restoration to normal anatomy, with a reduction in stromal edema, thickness and extracellular matrix deposition, coupled with improved epithelial morphology. The reduction in fibrotic markers by hrDecorin has been previously demonstrated across numerous animal models; modulating a range of growth factors (e.g., VEGF, IGF-1, EGF, PDGF) and their receptors, in particular TGFβ signaling via SMAD 2 and 3 pathways, preventing differentiation of corneal fibroblasts. In addition, its regulation of matrix metalloproteinase and tissue inhibitors of metalloproteinase results in fibrolysis and attenuated scar formation.^44,59–61^

**Fig. 6:**
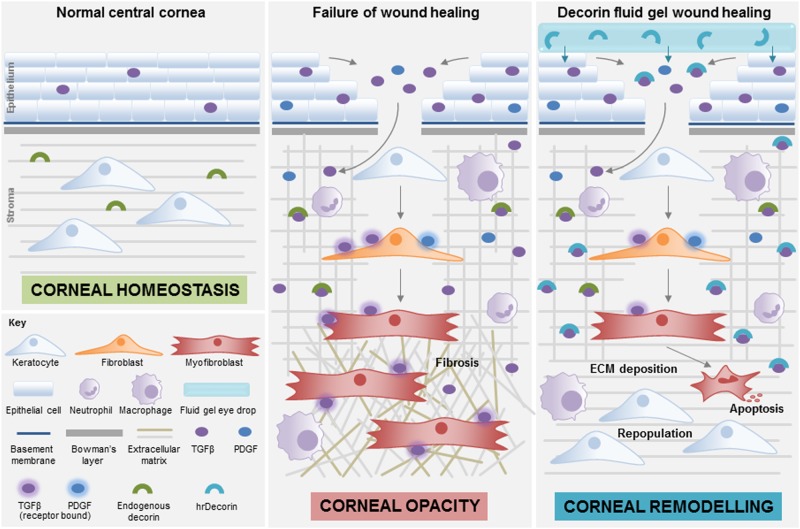
Mechanisms of corneal fibrosis and the effects of hrDecorin fluid gel. If corneal wound-healing fails, high concentrations of TGFβ result in the continued activation of myofibroblasts within the stroma leading to excessive ECM deposition, disorganized collagen, and inflammation resulting in corneal fibrosis. At this point, although endogenous decorin is present, it is unable to maintain corneal homeostasis. This may be due to insufficient concentrations to inhibit TGFβ hyperactivity or because the endogenous decorin is not freely available and bound within the ECM. Providing a sustained release of exogenous hrDecorin from the fluid gel helps to increase decorin levels and reduce TGFβ hyperactivity, thus providing a permissive environment for wound healing, corneal remodeling, and restoration of corneal integrity

The intrinsic ability of hrDecorin to aid healing, and in particular reduce scarring, has been enhanced by introducing the fluid gel carrier, improving retention time on the ocular surface. Given the severe extent of injury in this mouse model, we suggest that the endogenous levels of decorin may not have been sufficient to neutralize the TGFβ hyperactivity and subsequent fibrotic cascade to prevent corneal scarring. It is also possible that the endogenous decorin, located at the ocular surface within the tear film, within the cornea and the aqueous humor, is bound and not freely available to sequester the active TGFβ (Fig. 6).

A unique feature of both our formulations, i.e., the fluid gel eye drop and the hrDecorin fluid gel eye drop, was their ability to enhance the rate of re-epithelialization compared with standard care. This is central to the limitation of ocular damage as persistent epithelial defects lead to dysfunctional corneal metabolism, stromal melts, and perforation. The addition of hrDecorin in the fluid gel eye drop formulation reduced ECM accumulation more than the standard of care and the fluid gel alone and, this was associated with further reductions in opacity (compared with earlier time points) in this model. This suggests our hrDecorin fluid gel eye drop provided sufficient doses to prevent fibrosis and promote wound resolution in this model or altered the chemical structure of the fluid gel to improve the bandaging effect. The benefits of this fluid gel formulation have been clearly demonstrated in vivo, observed both physically, with a reduction in corneal opacity, and pharmacologically, relating to diminished fibrotic markers. However, owing to legislative constraints, the data generated within this study was limited to a 16 day time point and it would be of interest to examine later time points in future studies.

The effects of the fluid gel alone on the damaged corneal surface suggest an influence over the endogenous growth factors, an effect that is enhanced by the addition of hrDecorin. The fluid gel may aid corneal healing through several mechanisms: first, the unique viscoelastic properties of the fluid gel acts as a liquid that self-structures upon the ocular surface to form a semi-solid occlusive therapeutic dressing for unperturbed healing to take place; second, helical domains formed during the gelation of the fluid gel may provide a mimetic scaffold for endogenous decorin to bind, sequestering key growth factors, e.g., TGFβ and/or exogenously delivered hrDecorin; third, the fluid gel matrix, comprising primarily of water (99.1%), creates a gradient driven diffusion of cytokines away from the wound site, again resulting in a restoration of the natural equilibrium needed to prevent fibrosis.

We have seen two different responses in the presence of the fluid gel and the Decorin fluid gel, and in future studies, it will be important to tease apart the mechanism for each. We expect that the fluid gel alone is providing a protective barrier while potentially influencing inflammatory cell and fibroblast behavior in a manner that we do not yet understand. Importantly, the fluid gels are facilitating regeneration of the corneal epithelium leading to wound closure. We are hypothesizing that the fluid gel is affecting the limbal epithelium stem cell niche by promoting proliferation and differentiation, which may be dysregulated in the disease situation as well as providing a therapeutic bandage to aid stromal repair. However, in this study we did not have a hyaluronate or a carboxymethylcellulose only group to interrogate whether these ocular lubricant devices have a similar effect. Furthermore, the multifaceted actions of decorin such as inhibition of inflammation and angiogenesis and regulation of autophagy, in addition to sequestering TGFβ within the context of an ocular wound healing environment, need to be investigated further before translation into the clinic.

In conclusion, we have demonstrated that a novel eye drop technology can be used to provide sustained delivery of anti-fibrotic drugs like hrDecorin topically to the cornea in a clinically relevant murine model of fibrosis associated with bacterial keratitis. The eye drop enabled the hrDecorin to remain in contact with the surface of the eye for long enough and at sufficient titers to significantly reduce corneal scarring or altered the structure of the gel to provide better therapeutic bandaging. Furthermore, this study has demonstrated that the unloaded fluid gel also possesses healing effects in its own right, suggested to arise through its intrinsic material microstructure and subsequent properties. Not only do the material properties of the eye drop enhance anti-scarring drug retention times, but the user-friendly nature of the drops would be welcomed by patients, providing a simple treatment to prevent the scarring pathology that is prevalent after corneal infection. Having demonstrated successful reduction in corneal opacity and a reduction in markers commonly indicative of the scarring process, when compared with the current standard of care, this technology presents an ideal treatment option for patients with microbial keratitis, reducing the occurrence of visually significant corneal opacity and potentially eradicating the need for corrective surgical intervention. Given that transplant availability and the facilities for surgical intervention are often not available in the developing world, we believe that this technology could, in the future, help to save the sight of many patients.

## Materials and methods

### Study design

All studies were approved by the Universities of Birmingham, UK, and California, Irvine with all mouse studies maintained according to the ARRIVE guidelines and in accordance with the ARVO statement for the Use of Animals in Ophthalmic and Vision Research. The aim of this study was to explore the use of a novel fluid gel to deliver hrDecorin to the ocular surface in order to reduce corneal opacity and scarring post-bacterial keratitis. The study was split into three evaluation stages: (i) material properties relating to ease of eye drop application, (ii) in vitro assessment of bioactivity of the formulated hrDecorin, and (iii) anti-scarring efficacy of the fluid gel with/without hrDecorin in vivo, using a mouse model of *Pseudomonas keratitis* (once the eyes were sterilized after infection) in comparison with the current standard of care. The sample size (*n* = 6 per experimental group) was based on the resource equation as the effect size was unknown. All analyses were performed by observers masked to experimental groupings and mice were randomly assigned to both treatment and control groups.

#### Fluid gel (FG) and hrDecorin fluid gel (DecFG) production

##### Preparation of fluid gel eye drops

Fluid gels were produced by first dissolving low acyl gellan gum (Kelco gel CG LA, Azelis, UK) in deionized water. Gellan powder was added to deionized water at ambient temperature in the correct ratio to result in a 1% (w/v) solution. The sol was heated to 70 °C under agitation, on a hotplate equipped with a magnetic stirrer, until all the polymer had dissolved. Once dissolved, gellan sol was added to the cup of a rotational rheometer (AR-G2, TA Instruments, UK) equipped with cup and vane geometry (cup: 35 mm diameter, vane: 28 mm diameter). The system was then cooled to 40 °C. hrDecorin (Galacorin™; Catalent, USA) in phosphate-buffered saline (PBS) (4.76 mg/ml) and aqueous sodium chloride (0.2 M) was then added to result in final concentrations of 0.9% (w/v) gellan, 0.24 mg/ml hrDecorin and 10 mM NaCl. Following this, the mixture was cooled at a rate of 1 °C/min under shear (450/s) to a final temperature of 20 °C. The sample was then removed and stored at 4 °C until further use. In the case of fluid gels without hrDecorin, ratios were adjusted so that the final eye drop had a composition of 0.9% (w/v) gellan, 10 mM NaCl.

#### Material characterization of the fluid gel eye drops

##### Microscopy

For transmission microscopy samples were first diluted using polyethylene glycol 400 (PEG400) at a ratio of 1:4 (eye drop to PEG400). Following this, samples were analyzed using an Olympus FV3000. Images were processed using ImageJ (http://imagej.nih.gov/ij/; provided in the public domain by the National Institutes of Health, Bethesda, MD, USA).

For scanning electron microscopy samples were first prepared for lyophilizing by diluting gellan in deionized water in the same manner as for transmission microscopy to a ratio of 1:9. Samples were then rapidly frozen using liquid nitrogen and placed in a freeze drier overnight to leave a powder. Dried sample was then attached to a carbon stub and analyzed using a SEM.

##### Rheology

Viscosity profiles were obtained using an AR-G2 (TA Instruments, UK) rheometer equipped with sandblasted parallel plates (40 mm, 1 mm gap height) at 20 °C. An equilibrium of 2 min was used to ensure constant test temperature. Following this, time dependent ramps up and down were applied ranging from 0.1 to 600 /s (3 min sweep times). Recovery profiles were obtained using the same apparatus, under single frequency. The sample underwent rejuvenation by shearing at 600/s for 10 s. Following this, storage and loss (G’, G”, respectively) were monitored at 1 Hz, 0.5% strain. The crossover point was used as the point at which the sample started to act like a viscoelastic solid.

### hrDecorin release from the fluid gel

Levels of hrDecorin release from the gel were determined cumulatively, by placing 1 ml of the fluid gel containing hrDecorin in a six-well plate. Then 2 ml of Dulbecco's modification of Eagle medium was placed over the sample and the plates were incubated at 37 °C. At each time point the media was removed for measurement of hrDecorin and replaced with fresh media. Decorin release was quantified using an enzyme-linked immunosorbent assay (ELISA) specific for human Decorin (R&D systems, Minneapolis, USA) in accordance with the manufacturer’s protocol.

#### Collagen fibrillogenesis

For the dose–response curves, 75 μl of PBS was added to each well of a 96-well plate kept on ice. Varying hrDecorin doses were prepared by adding 400 μg/ml of hrDecorin to the first well and subsequently serial diluting (two-fold dilution) across the plate. Following dilution, a further 150 μl of PBS buffer was added to each well. Then, 75 μl of collagen type I (rat tail; Corning, UK) (800 μg/ml) was added to each well and incubated for 2 h at 37 °C. Subsequent absorbance readings were taken using a 405 nm plate reader. Each assay consisted of duplicate blank controls, and triplicate standard dilutions followed by triplicate sample dilutions. Kinetics of fibril formation were determined using a similar setup as the dose–response, without serial dilution; incubating the samples within the plate reader, and taking data points every 2 min.

### *P. keratitis* model and in vivo stereomicroscopy

**Fig. 7:**
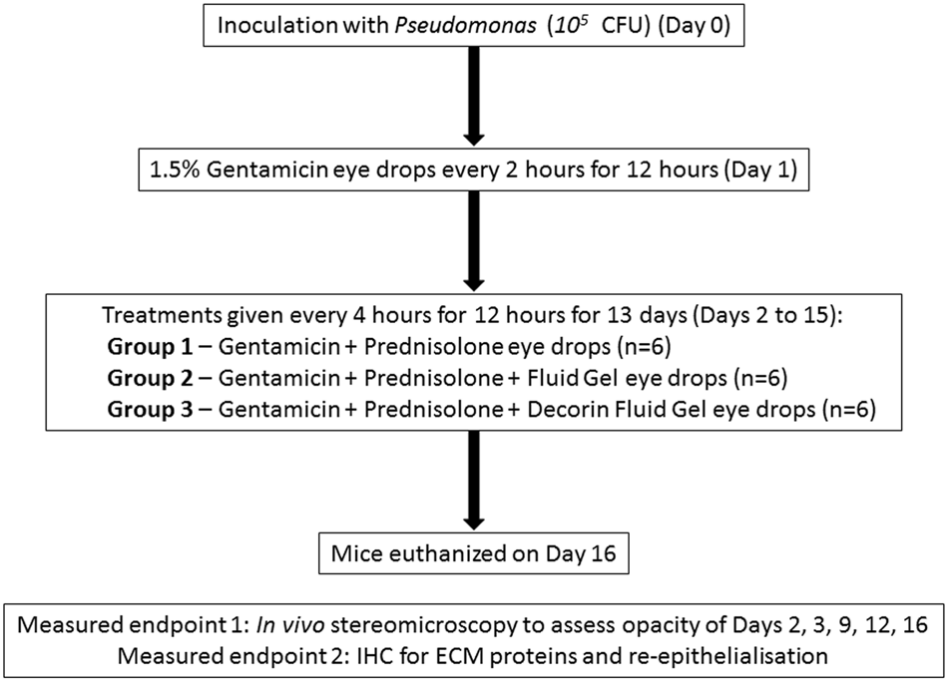
In vivo experimental design. Experimental design for the in vivo *Pseudomonas keratitis* study in which the fluid gel eye drops with and without hrDecorin were compared with Gentamicin and Prednisolone eye drops alone

The treatment administration regimes for the in vivo *Pseudomonas* model are shown in Fig. 7. Groups of naive intact and infected corneas taken at day 2 were also included in the experimental plan. The sample size of *n* = 6 for each control or treatment group was based on the resource equation^49^ as the effect size was unknown. Mice were randomly assigned to each treatment and control group before they were infected with *Pseudomonas*. Each treatment procedure and the sample sizes are described in further detail below. For the in vivo studies, analyses were performed by investigators masked to the experimental groups.

### In vivo murine model of *P. keratitis*

*P. aeruginosa* PAO1 strain was cultured in high salt Luria Bertani (10 g of tryptone, 5 g of yeast extract, and 11.7 g of NaCl per L, supplemented with 10 mM MgCl_2_ and 0.5 mM CaCl_2_) at 37 °C for 18 h. Sub-cultures were derived at an optic density (OD) of 0.2 (OD 650 nm approx. 1 × 10^8^ CFU/ml). *P. aeruginosa* were washed (3×) in PBS, centrifuged at 300 rpm for 5 min and re-suspended in PBS at a density of 1 × 10^5^ CFU/2.5 µl. C57BL/6 mice (Jackson Laboratory, CA, USA) were housed in pathogen-free conditions, given free access to food and water and were maintained according to the ARRIVE guidelines, the ARVO statement for the Use of Animals in Ophthalmic and Vision Research and also adhered to guidelines set out by the University of California, Irvine. For inoculation, mice were anaesthetized and one corneal epithelium was abraded with 3 × 1 mm parallel scratches using a 26 G needle and inoculated with 2.5 µl *P. aeruginosa* (1 × 10^5^ CFU) (strain PAO1)^64,65^. Mice remained sedated for 2 h post inoculation to permit penetration of the infection into the eye, and placed in recovery. After 24 h, conscious mice were treated with 5 µl of gentamicin (1.5%, QEHB Pharmacy, Birmingham, UK) every 2 h for a 12-hour period, to sterilize the infection. After a further 12 h, mice were administered eye drops (5 µl of each compound) every 4 h between 8 am and 8 pm for a further 13 days depending on their treatment group: (1) Gentamicin + Prednisolone (0.5%, QEHB Pharmacy), (2) Gentamicin + Prednisolone + fluid gel, or (3) Gentamicin + Prednisolone + fluid gel with hrDecorin. Mice were examined for corneal opacification, ulceration, and perforation. En-face 24-bit color photographs of the cornea were captured with a SPOT RTKE camera (Diagnostic Instruments) connected to a Leica MZF III stereo Microscope. Mice were killed by cervical dislocation under anesthetic at 16 days and eyes enucleated and placed in 4% paraformaldehyde (PFA) in PBS for processing for immunohistochemistry.

### Opacity quantification

Two masked independent clinical ophthalmologists analyzed all photographs in the same randomized order (the order was provided by an independent statistician). The area of opacification was delineated and measured in mm^2^ ± SEM using ImageJ. Definitions of corneal opacification, adequate, and inadequate images were agreed upon prior to commencement of image analysis by the observers. The randomized order dictated that there should be no time-trend in the measured areas. The limits of agreement between observers were assessed using the Bland–Altman method [62] on 99 paired opacity measurements. Differences in measurement were approximately normally distributed. The Bland–Altman analysis revealed that one assessor was likely to assess sizes to be slightly smaller than the other but the mean difference was not statistically different from zero (two-sided *p* value = 0.29). A small number of replicates of measurements within assessor were conducted but not enough to formally test the limits of agreement within observer.

### Tissue processing and immunohistochemistry for re-epithelialization and ECM

Enucleated eyes for immunochemistry (IHC) were post-fixed by immersion in 4% in PBS overnight at 4 °C before cryoprotection using increasing concentrations of sucrose in PBS (10, 20, and 30%; Sigma) for 24 h each at 4 °C. Eyes were then embedded in optimal cutting temperature embedding medium (Thermo Shandon, Runcorn, UK) in peel-away mold containers (Agar Scientific, Essex, UK) and later sectioned in the parasagittal plane at − 22 °C using a cryostat microtome (Bright, Huntingdon, UK) at a thickness of 15 μm, and placed onto Superfrost slides (Fisher Scientific, USA). Central sections (in the optic nerve plane) were used for all IHC studies and stored at − 80 °C. Frozen sections were left to thaw for 30 min before 3 × 5 min washings in PBS followed by a 20 min permeabilization with 0.1% Triton X-100 (Sigma). Non-specific antibody binding sites in tissue sections were blocked for 30 min using 0.5% bovine serum albumin (BSA), 0.3% Tween-20 (all from Sigma), and 15% normal goat serum (Vector Laboratories, Peterborough, UK) in PBS before incubating overnight in 4 °C in primary antibody (αSMA, Laminin and fibronectin; 1:200; all from Sigma) again followed by washing 3 × 5 min, and incubating for 1 h at room temperature with a secondary antibody (Goat anti-mouse Alexa Fluor 488 1:500, Goat anti-mouse Alexa Fluor 594 1:500, Molecular Probes, Paisley, UK). Sections were then washed for 3 × 5 min and mounted in Vectorshield mounting medium containing DAPI (Vector Laboratories). Control tissue sections incubated with secondary antibody alone were all negatively stained.

### Immunohistochemical imaging and quantification

After IHC, sections were imaged on a Zeiss Axioscanner fluorescent microscope (Axio Scan.Z1, Carl Zeiss Ltd.) at × 20 using the same exposure times for each antibody. IHC staining was quantified by measuring pixel intensity according to the methods previously described.^61^ In brief, the region of interest used for quantitation of ECM IR was defined by a region of interest which was same prescribed size for all eyes/treatments within the stroma. Each stroma had a total of 30 individual intensity measurements (regions of interest) taken to cover the whole area. ECM deposition was quantified within these defined regions of interests and the percentage of IR pixels above a standardized background threshold from intact corneas was calculated using ImageJ. For each antibody, the threshold level of brightness in the area of the stroma was set using intact untreated corneas to define the reference level for test group analysis. Images were assigned randomized file names to ensure masking of treatment groups for the assessor.

### Statistical analysis

All statistical analyses were performed using SPSS 20 (IBM, Chicago, IL, USA). Normal distribution tests were carried out to determine the most appropriate statistical analysis to compare treatments. Statistical significance was determined at *P* < 0.05. For opacity measurements, corneal width, epithelial thickness, αSMA, fibronectin, and laminin data were analyzed using analysis of variance with Tukey post hoc tests. For 4′,6-diamidino-2-phenylindole measurements of epithelial cell layer numbers, as the data were not normally distributed, a Kruskal–Wallis test was used.

## Supplementary information

Supplementary Figure 1

## Data Availability

The data sets generated during and/or analyzed during the current study are available from the corresponding author on reasonable request.

## Supplementary information

**Supplementary information** accompanies the paper on the *npj Regenerative Medicine* website (10.1038/s41536-018-0061-4).

## References

[1] Oliva MS, Schottman T, Gulati M (2012). Turning the tide of corneal blindness. Indian J. Ophthalmol..

[2] Konda N (2014). Microbial analyses of contact lens–associated microbial Keratitis. Optom. Vision. Sci..

[3] Stapleton F, Dart J, Seal D, Matheson M (1995). Epidemiology of Pseudomonas aeruginosa keratitis in contact lens wearers. Epidemiol. Infect..

[4] Wu YTY, Willcox M, Zhu H, Stapleton F (2015). Contact lens hygiene compliance and lens case contamination: a review. Contact Lens Anterior Eye.

[5] O’Brien T (2003). Management of bacterial keratitis: beyond exorcism towards consideration of organism and host factors. Eye.

[6] Willcox MD (2007). Pseudomonas aeruginosa infection and inflammation during contact lens wear: a review. Optom. Vis. Sci..

[7] Bukowiecki A, Hos D, Cursiefen C, Eming SA (2017). Wound-healing studies in cornea and skin: parallels, differences and opportunities. Int. J. Mol. Sci..

[8] Dua HS, Shanmuganathan VA, Powell-Richards AO, Tighe PJ, Joseph A (2005). Limbal epithelial crypts: a novel anatomical structure and a putative limbal stem cell niche. Br. J. Ophthalmol..

[9] Wilson SE (2002). Analysis of the keratocyte apoptosis, keratocyte proliferation, and myofibroblast transformation responses after photorefractive keratectomy and laser in situ keratomileusis. Trans. Am. Ophthalmol. Soc..

[10] Jester JV, Brown D, Pappa A, Vasiliou V (2012). Myofibroblast differentiation modulates keratocyte crystallin protein expression, concentration, and cellular light scattering. Invest. Ophthalmol. Vis. Sci..

[11] Torricelli AAM, Santhanam A, Wu J, Singh V, Wilson SE (2016). The corneal fibrosis response to epithelial-stromal injury. Exp. Eye Res..

[12] Tandon A, Tovey JC, Sharma A, Gupta R, Mohan RR (2010). Role of transforming growth factor Beta in corneal function, biology and pathology. Curr. Mol. Med..

[13] Stramer BM, Austin JS, Roberts AB, Fini ME (2005). Selective reduction of fibrotic markers in repairing corneas of mice deficient in Smad3. J. Cell Physiol..

[14] Wilson SE (2012). Corneal myofibroblast biology and pathobiology: generation, persistence, and transparency. Exp. Eye Res..

[15] Stramer BM, Zieske JD, Jung JC, Austin JS, Fini ME (2003). Molecular mechanisms controlling the fibrotic repair phenotype in cornea: implications for surgical outcomes. Invest. Ophthalmol. Vis. Sci..

[16] Torricelli AA, Singh V, Santhiago MR, Wilson SE (2013). The corneal epithelial basement membrane: structure, function, and disease. Invest. Ophthalmol. Vis. Sci..

[17] Allan BD, Dart JK (1995). Strategies for the management of microbial keratitis. Br. J. Ophthalmol..

[18] Gokhale NS (2008). Medical management approach to infectious keratitis. Indian J. Ophthalmol..

[19] Ralph RA (2000). Tetracyclines and the treatment of corneal stromal ulceration: a review. Cornea.

[20] Shoham A, Hadziahmetovic M, Dunaief JL, Mydlarski MB, Schipper HM (2008). Oxidative stress in diseases of the human cornea. Free Radic. Biol. Med..

[21] Dua HS (1999). Amniotic membrane transplantation. Br. J. Ophthalmol..

[22] Mamede, A. C. & Botelho, M. F. *Amniotic Membrane: Origin, Characterization and Medical Applications.* (Springer: Netherlands, 2015).

[23] Sorsby A, Haythorne J, Reed H (1947). Further experience with amniotic membrane grafts in caustic burns of the eye. Br. J. Ophthalmol..

[24] Gabler B, Lohmann CP (2000). Hypopyon after repeated transplantation of human amniotic membrane onto the corneal surface. Ophthalmology.

[25] Gauthier AS (2017). Corneal transplantation: study of the data of a regional eye bank for the year 2013 and analysis of the evolution of the adverse events reported in France since 2010. Cell Tissue Bank.

[26] Nguyen P, Rue K, Heur M, Yiu SC (2014). Ocular surface rehabilitation: application of human amniotic membrane in high-risk penetrating keratoplasties. Saudi J. Ophthalmol..

[27] Qazi Y, Hamrah P (2013). Corneal allograft rejection: immunopathogenesis to therapeutics. J. Clin. Cell Immunol..

[28] Thom SB (1997). Effect of topical anti-transforming growth factor-β on corneal stromal haze after photorefractive keratectomy in rabbits. J. Cataract Refract. Surg..

[29] Cordeiro MF, Gay JA, Khaw PT (1999). Human anti-transforming growth factor-β2 antibody: a new glaucoma anti- scarring agent. Invest. Ophthalmol. Vis. Sci..

[30] Jester JV, Huang J, Petroll WM, Cavanagh HD (2002). TGFβ induced myofibroblast differentiation of rabbit keratocytes requires synergistic TGFβ, PDGF and integrin signaling. Exp. Eye Res..

[31] Mohan RR, CK Tovey J, Gupta R, Sharma A, Tandon A (2011). Decorin biology, expression, function and therapy in the cornea. Curr. Mol. Med..

[32] Du S, Wang S, Wu Q, Hu J, Li T (2013). Decorin inhibits angiogenic potential of choroid-retinal endothelial cells by downregulating hypoxia-induced Met, Rac1, HIF-1α and VEGF expression in cocultured retinal pigment epithelial cells. Exp. Eye Res..

[33] Grant DS (2002). Decorin suppresses tumor cell-mediated angiogenesis. Oncogene.

[34] Iozzo RV (2011). Decorin antagonizes IGF receptor I (IGF-IR) function by interfering with IGF-IR activity and attenuating downstream signaling. J. Biol. Chem..

[35] Mohan RR (2011). Decorin gene therapy delivered with adeno-associated virus effectively retards corneal neovascularization in vivo. PLoS ONE.

[36] Kalamajski S, Oldberg Aring (2010). The role of small leucine-rich proteoglycans in collagen fibrillogenesis. Matrix Biol..

[37] Nakamura N (2000). Decorin antisense gene therapy improves functional healing of early rabbit ligament scar with enhanced collagen fibrillogenesis in vivo. J. Orthop. Res..

[38] Reed CC, Iozzo RV (2002). The role of decorin in collagen fibrillogenesis and skin homeostasis. Glycoconj. J..

[39] Zhang G (2009). Genetic evidence for the coordinated regulation of collagen fibrillogenesis in the cornea by decorin and biglycan. J. Biol. Chem..

[40] Bredrup C, Knappskog PM, Majewski J, Rødahl E, Boman H (2005). Congenital stromal dystrophy of the cornea caused by a mutation in the decorin gene. Invest. Ophthalmol. Vis. Sci..

[41] Isaka Y (1996). Gene therapy by skeletal muscle expression of decorin prevents fibrotic disease in rat kidney. Nat. Med..

[42] Margetts PJ (2002). Antiangiogenic and antifibrotic gene therapy in a chronic infusion model of peritoneal dialysis in rats. J. Am. Soc. Nephrol..

[43] Stander M, Naumann U, Wick W, Weller M (1999). Transforming growth factor-beta and p-21: multiple molecular targets of decorin-mediated suppression of neoplastic growth. Cell Tissue Res..

[44] Ahmed Z (2014). Decorin blocks scarring and cystic cavitation in acute and induces scar dissolution in chronic spinal cord wounds. Neurobiol. Dis..

[45] Logan A, Baird A, Berry M (1999). Decorin attenuates gliotic scar formation in the rat cerebral hemisphere. Exp. Neurol..

[46] Logan A, Frautschy SA, Gonzalez AM, Sporn MB, Baird A (1992). Enhanced expression of transforming growth factor β1 in the rat brain after a localized cerebral injury. Brain Res..

[47] Rathore K, Nema R (2009). An insight into ophthalmic drug delivery system. Int J. Pharm. Sci. Drug Res..

[48] Snibson G (1992). Ocular surface residence times of artificial tear solutions. Cornea.

[49] Norton I, Jarvis D, Foster T (1999). A molecular model for the formation and properties of fluid gels. Int. J. Biol. Macromol..

[50] Wolf B, Frith WJ, Singleton S, Tassieri M, Norton IT (2001). Shear behaviour of biopolymer suspensions with spheroidal and cylindrical particles. Rheol. Acta.

[51] Barnes H. A. A handbook of elementary rheology. Institute of Non-Newtonian Fluid Mechanics. p 5–130 (University of Wales, Ab-erystwyth, Wales, 2000).

[52] Graessley W. W. The entanglement concept in polymer rheology. The entanglement concept in polymer rheology. p 1–179 (Springer, Berlin, 1974).

[53] Watanabe H (1999). Viscoelasticity and dynamics of entangled polymers. Prog. Polym. Sci..

[54] Winter H (1987). Can the gel point of a cross linking polymer be detected by the G′–G ″crossover?. Polym. Eng. Sci..

[55] Karmakar M, Sun Y, Hise AG, Rietsch A, Pearlman E (2012). Cutting edge: IL-1β processing during Pseudomonas aeruginosa infection is mediated by neutrophil serine proteases and is independent of NLRC4 and caspase-1. J. Immunol..

[56] Mishima S, Gasset A, Klyce S, Baum J (1966). Determination of tear volume and tear flow. Invest. Ophthalmol. Vis. Sci..

[57] Sun Y (2010). TLR4 and TLR5 on corneal acrophages regulate pseudomonas aeruginosa Keratitis by signaling through MyD88-dependent and -independent pathways. J. Immunol..

[58] McClintic SM (2013). Improvement in corneal scarring following bacterial keratitis. Eye.

[59] Botfield H (2013). Decorin prevents the development of juvenile communicating hydrocephalus. Brain.

[60] Grisanti S (2005). Decorin modulates wound healing in experimental glaucoma filtration surgery: a pilot study. Invest. Ophthalmol. & Vis. Sci..

[61] Hill LJ (2015). Decorin reduces intraocular pressure and retinal ganglion cell loss in rodents through fibrolysis of the scarred trabecular meshwork. Invest. Ophthalmol. Vis. Sci..

[62] Bland JM, Altman DG (1999). Measuring agreement in method comparison studies. Stat. Methods Med. Res..

