# Supplementary material for: Sustained release of decorin to the surface of the eye enables scarless corneal regeneration
Source: NPJ Regen Med. 2018 Dec 21;3:23. doi: 10.1038/s41536-018-0061-4 (PMC6303295; doi:10.1038/s41536-018-0061-4)
Supplement: Supplementary file 1 — Supplementary Figure 1 [file 41536_2018_61_MOESM1_ESM.docx]

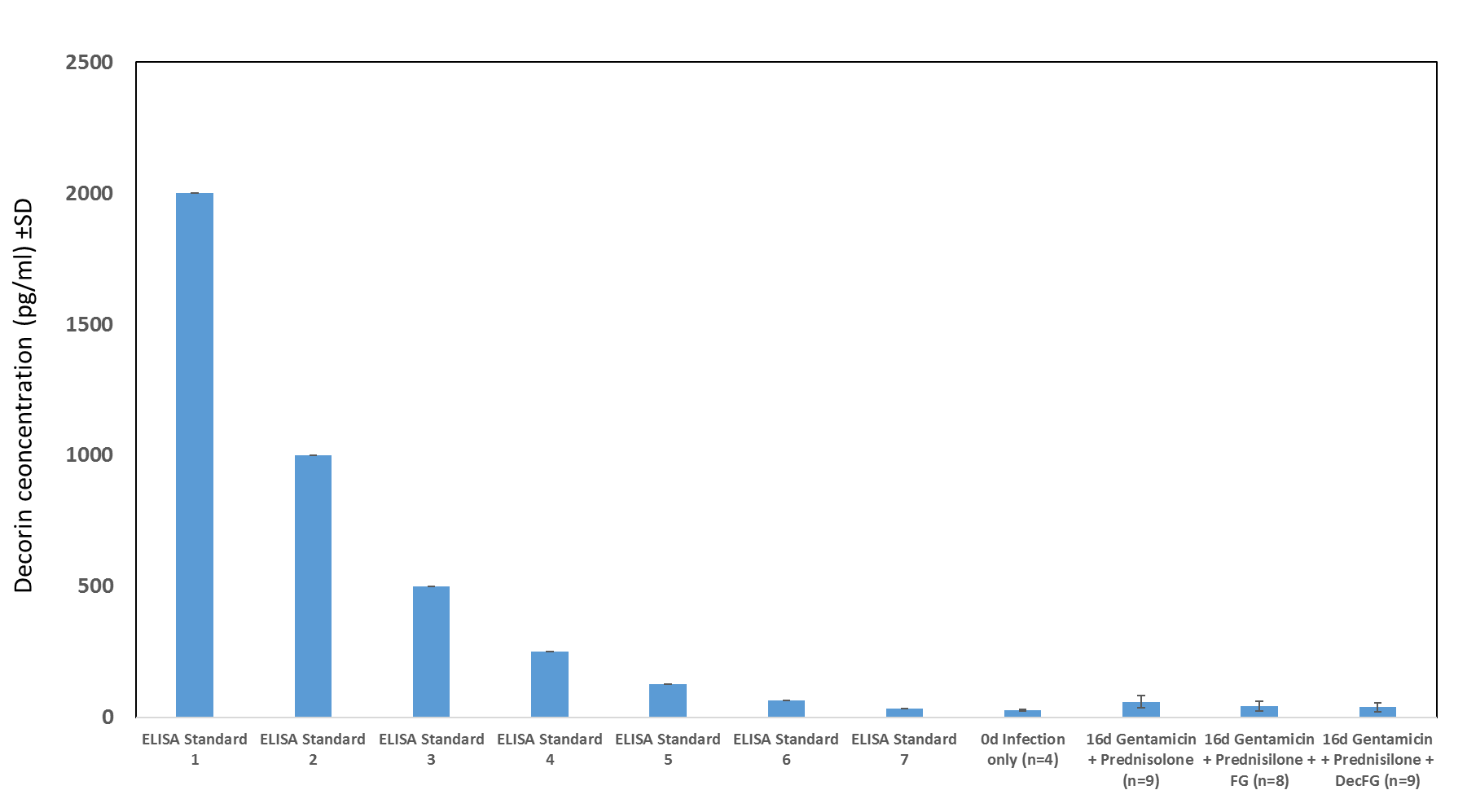


Supplementary Figure 1. Mouse serum decorin levels. Concentrations of decorin in mouse serum taken from mice with infection only, n=4, and after eye drop treatments with Gentamicin + Prednisolone, n=9, Gentamicin + Prednisolone + FG, n=8 and Gentamicin + Prednisolone + Dec FG, n=9.
